# Integrating Evolutionary, Cultural, and Computational Psychiatry: A Multilevel Systemic Approach

**DOI:** 10.3389/fpsyt.2022.763380

**Published:** 2022-04-04

**Authors:** Axel Constant, Paul Badcock, Karl Friston, Laurence J. Kirmayer

**Affiliations:** ^1^Department of Philosophy, The University of Sydney, Darlington, NSW, Australia; ^2^Centre for Youth Mental Health, The University of Melbourne, Parkville, VIC, Australia; ^3^Orygen, Parkville, VIC, Australia; ^4^Wellcome Centre for Human Neuroimaging, University College London, London, United Kingdom; ^5^Department of Psychiatry, McGill University, Montréal, QC, Canada

**Keywords:** cultural psychiatry, computational psychiatry, computational phenotyping, major depressive disorder (MDD), evolutionary psychiatry

## Abstract

This paper proposes an integrative perspective on evolutionary, cultural and computational approaches to psychiatry. These three approaches attempt to frame mental disorders as multiscale entities and offer modes of explanations and modeling strategies that can inform clinical practice. Although each of these perspectives involves systemic thinking, each is limited in its ability to address the complex developmental trajectories and larger social systemic interactions that lead to mental disorders. Inspired by computational modeling in theoretical biology, this paper aims to integrate the modes of explanation offered by evolutionary, cultural and computational psychiatry in a multilevel systemic perspective. We apply the resulting Evolutionary, Cultural and Computational (ECC) model to Major Depressive Disorder (MDD) to illustrate how this integrative approach can guide research and practice in psychiatry.

## Introduction

### The Problem of Disciplinary Boundaries

Contemporary psychiatry assumes that gene–environment interactions over the course of developmental trajectories contribute to the etiology of mental disorders (1). These trajectories depend on processes at multiple levels, including epigenetic, neurophysiological, behaviors and interpersonal interactions, which are embedded in larger social systemic contexts. Our currently limited knowledge about such interactions is a challenge for efforts to ground diagnostic nosology and clinical practice in a mechanistic understanding of the relations between multiple levels that constitute the complex pathways to mental disorders (2). The aim of this paper is to advance an integrative perspective that bridges three theoretical domains in psychiatry, which taken together, promise a mechanistic^1^ understanding of the systemic processes and trajectories that underwrite psychopathology: evolutionary psychiatry, cultural psychiatry, and computational psychiatry.

Over the last 40 years, evolutionary, cultural, and computational psychiatry have each developed theoretical, empirical and clinical approaches to psychopathology. Although representing different conceptual models and research methodologies, all three approaches aim to advance non-reductionist, mechanistic, and multilevel account of the pathways to mental disorders. As the name suggests, *evolutionary psychiatry* endeavors to explain mental disorders in terms of the evolutionary and genetic origins of the phenotypic traits (12). *Cultural psychiatry* emphasizes the role of culturally mediated social practices in development and the circular causality between illness behavior and social context (13). Finally, the emerging field of *computational psychiatry* studies failures in decision-making and dysfunctional behavior using multi-level computational models (14).

Despite some recent exceptions (15, 16), each approach has remained largely siloed. This lack of dialogue results from institutional and conceptual difficulties in crossing disciplinary boundaries (17). Disciplinary boundaries are the consequence of particular research histories and traditions but also reflect specific scientific ontologies (18). Ontologies underwrite research agendas (19), which reflect researchers' beliefs about what questions science should address and what kind of answers are satisfying (20), and that lead researchers to operate under different “thought styles” (21). Disciplinary ontologies require that researchers become skilled in using specific methods, which render measurable and ontologically “real” or conceivable certain dimensions of the object of inquiry (22). By the same token, due to constraints of time and resources, commitments to disciplinary ontologies also limit researchers' skills and impede the study of certain dimensions of phenomena and may make them invisible or even inconceivable. The result then is progress on some fronts but lack of attention to other, possibly crucial, facets or dimensions. This effect of disciplinary ontologies is especially concerning in the context of psychiatry, which is concerned with human problems that clearly involve multiple processes that affect physiology, behavior and experience (23). Advancing an integrative perspective, requires some way to move beyond these disciplinary blinders. We propose that unifying cultural, evolutionary and computational psychiatry can enable significant strides toward an integrative view.

### The Scope of the Integrative Perspective

This paper starts with an overview that lays out some assumptions and methodological strategies employed in evolutionary, cultural, and computational psychiatry (§2). We will not discuss evolutionary, cultural or computational psychiatry in their entirety. Rather, we focus on key aspects of these approaches—mainly modes of reasoning about mental disorders—that could be merged through an interdisciplinary way of thinking about mental disorders.

#### Key Aspects of Evolutionary Psychiatry

With respect to evolutionary psychiatry, we will focus on adaptationist reasoning about pathological mental traits, which can be distinguished from population genetics thinking (24). Adaptationist reasoning in evolutionary psychiatry emphasizes the role of natural selection when making sense of mental traits observed in clinical settings (25); population genetics thinking may be viewed as a research driven attempt at explaining changes in the genetic makeup of a population and the preservation of alleles that contribute to certain mental disorders (24). The distinction between population genetics thinking and what we call adaptationist thinking can be framed more generally in terms of what some historians have identified as the distinction between the modern synthesis and the ethological perspective (26). This division is interesting in that it carves out two interconnected questions about mental disorders that are approached with distinct reasoning patterns. The ethological perspective asks, “How can we understand mental disorders as traits that have evolved in humans and other animal species to serve certain functions?” and seeks answers based on the relation between phenotype (e.g., behavior) survival value and fitness. In turn, the modern synthesis perspective asks, “How can we explain the preservation of alleles underlying mental disorders in a population?” and seeks answers based on a wide array of evolutionary mechanisms, including, though not limited to, the logic of survival and fitness under natural selection (e.g., drift, mutations, and gene flow). The extension of the modern synthesis—the extended evolutionary synthesis (27)—suggests supplementing the mechanisms of evolution with channels of inheritance and processes that are external to the organism (e.g., cultural inheritance, niche construction, and development). The adaptationist rationale—on which we focus—can be assimilated to the ethological perspective.

#### Key Aspects of Cultural Psychiatry

With respect to cultural psychiatry, we will focus on how cultural context may shape mental disorders through a variety of intra- and interpersonal feedback loops, including what Hacking has termed “the looping effect of human kinds” (19). Cultural psychiatry studies the ways in which culture and social context shape the etiology (causes), phenomenology (experience), clinical presentation (expression), and trajectory of mental disorders (28). This includes the person's own modes of self-construal and the responses of others, which draw from cultural narratives, models and metaphors. Taken together these constitute the ontology of a mental disorder. Although this will not be our focus here, it is important to note that cultural psychiatry also leverages the notion of culture to orient clinical assessment, treatment, and prevention (e.g., situating illness experience in its social and cultural context to identify the significance of cultural expressions of distress and their impact on the course and outcome of mental health problems) (29). Cultural psychiatry also emphasizes self-reflexive practice, through studies that reveal the cultural assumptions of the institutions of psychiatry itself (e.g., ethnocentric biases) that may affect mental health research and clinical practice as well as illness experience (30, 31).

#### Key Aspects of Computational Psychiatry

With respect to computational psychiatry, we will focus on the rationale of modeling psychiatry. Computational psychiatry involves the use of algorithmic methods to model and analyze clinical and behavioral data (32). This includes two broad, though interrelated lines of work in computational psychiatry: (i) *data-driven* computational psychiatry, involving the use of artificial intelligence and machine learning with large datasets (“big data”) to develop more precise characterizations of patients that have some predictive validity in relation to treatment response and course of illness; and (ii) *theory-driven* computational modeling, which develops biologically plausible accounts of neural processing that can explain particular forms of psychopathology (33). The focus here will be on the latter approach, which aims to understand the mechanisms of psychiatric disorders by constructing computational models.

Our proposed integration of cultural, evolutionary and computational psychiatry aims to show how adaptationist thinking and the social-cultural notion of looping effects can be integrated using the methods of modeling psychiatry. To illustrate the potential of this integration, we describe a generic model for the study of mental disorders that inherits principles of evolutionary and cultural psychiatry (§3). The hope is that the resulting Evolutionary Computational Cultural (ECC) model will exemplify the interdisciplinary approach we advocate. The end of part three illustrates an application of this model using the clinical example of Major Depressive Disorder (MDD).

## Evolution, Culture, and Computation in Psychiatry

The difference in disciplinary ontologies poses a central theoretical challenge for collaboration among evolutionary, cultural and computational psychiatry. How can we think through the ideas of the evolutionary approach in computational terms; of cultural ideas in evolutionary terms; or computational ideas in cultural terms? What do we need to know to map one theoretical construct onto the other and what concepts and relations require special attention? This process of inter-theoretic mapping needs to start with a general understanding of principles employed in evolutionary, cultural and computational psychiatry. We will now consider adaptationist thinking, the ontology of mental disorders, and modeling psychiatry.

### Evolutionary Psychiatry

Medicine often employs functional models of health and diseases based on principles of human physiology. These models indicate how the body is supposed to function. Pathology can then be identified as a disruption or impairment of this function (34). For instance, we assume that the heart is designed to pump blood; and this is why, no matter the cause, congestive heart failure may be confidently described as a malfunction (35, 36). Although efforts have been made to define mental dysfunction in a similar way (37, 38), this effort has been impeded by the fact that the human mind has multiple functions that depend on adaptive context. Attempts to characterize brain function are intensively debated (39). One consequence of this lack of clarity about the functions of mind and brain is difficulty in distinguishing between disorders and protective responses (35). For instance, we know that congestive heart disease is a disorder and that fever is a protective response, because the former can be said to result from a failure of a function of the heart (e.g., pumping blood), whereas the latter reflects a functional biological response to infection (35).

Evolutionary psychiatry has sought to address this limitation by exploring plausible functions of mind and brain against the backdrop of human evolution. By applying the principles of evolutionary biology and psychology, evolutionary psychiatry aims to provide a basis to distinguish normal and pathological mental functioning, based on the notion of adaptive fitness (40). This leads to a view of mental disorders as “harmful dysfunctions” (41). As will be detailed below, in the account of mental disorders as “harmful dysfunctions”, the dysfunction refers to the functional aspect of the proximal mechanism (e.g., regulation of dopamine signaling), whereas the failure is defined in terms of discrepancies with respect to the way that mechanism ought to function from an evolutionary point of view (e.g., regulation sufficient to enable an adaptive response to the environment). In turn, the “harmful” component refers to value-laden terms that are often qualifiers of the disorder (e.g., autistic individuals' “lack of motivation”).

The problems that surround Wakefield's concept of mental disorder are at least two-fold (42). First, there is the problem of identifying the evolutionary adaptive process against which the dysfunctional mechanism can be evaluated: this has been termed “the problem of evolutionary function”. Second, there is the problem of the scientific validity of the notion of “harmful”, which is generally recognized to be, at least partially, socially and historically contingent. Indeed, according to the view of harmful dysfunction theory, although value-laden qualifiers are an essential part of the definition of mental disorders, the study of their functional role is difficult to assimilate to a purely evolutionary view. Yet, as argued by cultural psychiatry, unpacking the meaning of “harm” and other evaluative qualifiers is essential since psychiatric disorders are both biological and social constructs that always occur in particular cultural contexts. This article will focus on the latter problem. In the section on Cultural Psychiatry, integrating a cultural approach will allow us to address this problem by providing a more complete view of the mechanisms of mental disorders that explicitly incorporates humanly constructed contexts and corresponding social interactions.

#### Defining Mental Disorders With Proximate and Ultimate Thinking

Evolutionary psychiatry proposes a research heuristic for the study of mental ill-health, organized around the question of “why did evolution leave us with traits that make us vulnerable to mental disorders?” (43). This framework integrates proximate (e.g., developmental) and ultimate (i.e., evolutionary) levels of causation when defining mental disorders [for a summary see: (44)]. Sciences that study proximal mechanisms typically answer questions of the form “how does it work?”, (e.g., “how does experience-dependent neuroplasticity operate?”), whereas sciences that study ultimate causes answer evolutionary questions of the form “why does it work?”; (e.g., “why has experience dependent plasticity been preserved throughout human evolutionary history?”) (45).

Evolutionary psychiatry defines mental disorders as dysfunctions of adaptive systems (or consequences of adaptive systems that are maladaptive in a new niche or context), and explains disorders in terms of vulnerabilities aggravated by developmental demands. Note, however, that this type of explanation remains controversial (46). Some mental disorders have been viewed as adaptive dysfunctions, that is, as adaptations *per se* [e.g., psychopathy as an adaptive strategy from a game theoretic point of view (47)]. In this review, we will not pursue the view of mental disorders as adaptive dysfunctions. Rather, we will focus on explanations in terms of aggravated vulnerabilities. The integration of proximate and ultimate causes allows evolutionary psychiatry to study the impact of evolutionary pathways on the nature of mental disorders and their expression over the lifespan. The proximate part of this view describes the workings of the specific mechanisms underlying the development of pathology and their expression in symptomatology, suffering or functional impairment. Conversely, the explanation in terms of ultimate causes involves relationships between mechanisms and traits (and their associated vulnerabilities) that are conserved over evolutionary history (48). In short, integrating proximate and ultimate causes allows evolutionary psychiatry to explain psychiatric conditions from the point of view of vulnerabilities stemming from phylogenetically old traits (49).

Proximate and ultimate thinking in psychiatry tends to operate under two interrelated modes of evolutionary thinking: adaptationism and population genetics. Of course, the distinction between evolutionary influences that constitute proximate and ultimate causes is made for epistemological reasons. A more fine-grained assessment of causality would consider phenomena across multiple spatiotemporal scales, ranging from biochemical to evolutionary, including the scales of individual developmental trajectories and of the coevolution of the human brain and our cultural niches (50). The strategy of dividing causality into proximate and ultimate causes allows us to distinguish phenomena about which we can meaningfully ask questions like “Why has it evolved to work that way?” from phenomena about which we would better ask “How does it work?”. For instance, ultimate causes may capture phenomena that unfold on a historical timescale for which answers to “how” questions will likely remain uncertain (e.g., “What were the exact mechanisms at play in the evolution of this population?”), and for which the response to a “Why” question may be preferred (e.g., “What principles of evolution can explain why this feature might emerge?”).

#### Adaptationist Thinking

One popular strategy for the study of evolutionary pathways to mental disorders is the adaptationist approach (51), which relies on the notion that evolution favors the replication of variations that lead to reproductive success (fitness). Since differential reproductive success is correlated with being adapted to environmental stressors, the genetic material passed onto offspring should lead to phenotypic traits that will be adapted, or well “designed”, to respond to these stressors (52). As applied in evolutionary psychiatry, adaptationism relies on the idea that vulnerabilities are shaped by Darwinian selection. Typically, it is not that natural selection selects “for” disorders (e.g., viewing disorders as affording some fitness advantages) (53). Rather, ultimate causes must be viewed as shaping genetic traits that may be expressed as suboptimal traits or vulnerabilities under certain proximate, developmental conditions (48). Put another way, the maintenance of “any suboptimality [or vulnerability] of a part is explained as its contribution to the best possible design for the whole” [(54), p. 586]. Again, the question is not “how do genes that predispose to a mental disorder provide a selective advantage?” (55) nor is the question directly “how do genes that predispose to a mental disorder persist?”. Rather, the question is “why are we vulnerable to some mental disorders?”, the answer to which explains the clinical presentation of the mental disorder in the current context. This is important because explanations in psychiatry should be explanations of mental disorders, not only explanations of their underlying biology. As we will see with cultural psychiatry, mental disorders are entities configured at the level of human agency and subjectivity. Inquiring how aspects of a person's biology make that person vulnerable to a mental disorder is usually more immediately relevant to clinical practice than exploring the evolutionary origin of that biology.

Darwinian rationales have been used to explain different pathways to mental disorders in terms of the maintenance of vulnerabilities in human evolutionary history (ultimate cause) enabled by developmental context (proximate cause). Box 1 summarizes some of the popular rationales in adaptationist accounts of medicine in general. Darwinian rationales have been applied to explaining mental disorders such as anxiety, phobic, delusional, stress-related and depressive disorders among other mental health problems (64–67). Importantly, all of these approaches assume the embeddedness of the individual in a larger systemic context. For instance, following a Darwinian rationale, the social risk hypothesis of depression (15, 68, 69) argues that normative symptoms of depression—triggered by social uncertainty—form an adaptive biobehavioral strategy that might have been selected to ensure the restabilization of individuals' social networks. Here, depression is thought to reduce socio-environmental volatility *via* three broad classes of action: it increases an individual's cognitive sensitivity to social risks; it reduces her propensity to engage in social behaviors with uncertain outcomes; and it promotes social signaling behaviors to elicit interpersonal support and defuse competitive encounters (e.g., reassurance seeking). When these responses fail to alleviate social stress (e.g., signaling fails to increase interpersonal support), depressive symptoms endure, and the individual can spiral into more severe and persistent distress that is recognized as clinical depression. To account for the prevalence of depression in a given population, from an epidemiological perspective, one could couple the social risk hypothesis with an evolutionary mismatch rationale (see Box 1) to explain why depression may increase in a society in which people tend to have sparse human social networks.

Box 1 **Adaptationist explanations for psychopathology.**

**Mismatch:** Vulnerabilities may emerge from differential rates in evolution that generate mismatches between the cultural developmental environment and evolutionarily old dispositions (e.g., disordered eating patterns leading to obesity, because of humans' tendency to seek energy-rich, sugary and fatty foods that were scarce in our ancestors' environment but that are now abundant) (56). A mismatch happens when the rate of change of environmental stressors exceeds the rate of change of individuals' adaptation. Depending on the scale at which the mechanism of adaptation lags behind, a mismatch will either be defined as *developmental*—i.e., a body-environment mismatch (57); or *evolutionary*—i.e., a genotype-environment mismatch (58, 59). Developmental mismatches are assumed to impair realized fitness (i.e., individuals' reproductive success), whereas evolutionary mismatches are assumed to impair the ability to achieve expected fitness (i.e., the sum of reproductive success weighted by fitness across all possible environments).

**Constraints:** Constraints on selection arise when the cost of adapting a vulnerability through natural selection is higher than the cost of preserving that vulnerability in the population. For instance, the cost of delivering human infants through the pelvis, although painful and often dangerous, does not outweigh the cost of reengineering the birth canal (48). “Rule of thumb” logical reasoning outweighs its cost in terms of logical errors (60); and Huntington's disease has a limited cost since its symptoms do not appear before the age of child-bearing (61).

**Trade-offs:** Trade-offs also favor the selection of vulnerabilities understood as defenses, according to “smoke detector” explanations (62). Smoke detector explanations apply in cases where it is more cost efficient to select for genes that result in traits likely to trigger false alarms than to fail to detect threat (e.g., predator or fatal pathogen). For instance, acute sensitivity to anxiety provoking situations increases the success of fight or flight responses (and thereby contributes to reproductive success), but it increases vulnerability to anxiety disorders. Similarly, fever is a defense against infection (63) but it may increase to the point of causing seizures

#### Limits and Prospects of Adaptationist Rationales

Adaptationist accounts explain mental disorders in terms of the vulnerabilities of systems that evolved to serve an adaptive function (e.g., depressive symptoms are an adaptive vulnerability whose function is to reconsolidate social networks but that can spiral into maladaptive responses). This makes an explicit link between normal functioning and pathology and provides a rationale for research with animal models that involve similar biobehavioral systems (70); hence the ties of adaptationist thinking with the ethological perspective. Adaptationism has been critiqued, however, on methodological and conceptual grounds (67), among others, on the fact that traits may persist and lead to vulnerabilities through processes other than selection (71). Indeed, there are many cases that cannot be explained solely based on Darwinian thinking. For instance, disorders such as schizophrenia, bipolar disorder, eating disorders, and obsessive-compulsive disorder are known to impair reproductive success (24). All things being equal in the world of natural selection, genetic variants predisposing individuals to such disorders (e.g., genetic vulnerabilities) should have been eliminated from the gene pool long ago. To explain pathways to mental disorders based on traits that have no obvious adaptive value, evolutionary accounts of mental disorders can go beyond the adaptationist narrative by appealing to other population-level phenomena.

Explanations based on population genetic thinking provide a complement to Darwinian explanations [for a review see: (24)]. For instance, processes of *balancing selection* can maintain multiple variations of alleles in the same gene (i.e., polymorphism) whose net fitness effects balance each other out, depending on the genetic or environmental context (72). Balancing selection requires that all the alleles involved have roughly equivalent fitness, and that some mechanisms countered the normal loss of these alleles due to drift. A good example of a balancing selection process is frequency dependent selection, where the fitness of some unit (e.g., allele AA) or trait depends on its frequency in a population [e.g., the hawk-dove situation (73)]. Frequency dependent selection might explain the maintenance of allelic susceptibility to psychopathy, as people with psychopathy would gain a fitness advantage in a population where the allele is rare and becomes disadvantageous when frequent because of anti-cheater vigilance (53, 74, 75). Like adaptationist rationales, rationales from population genetics explain the persistence of dysfunctional genetic variations (e.g., vulnerabilities to illness) that would normally impair evolutionary success. This provides evolutionary psychiatry with a functional model of mental health and disease based on biological principles. It is important to note that there are many other population genetics models that can explain the persistence of harmful variations (24). The example of balancing selection is introduced here to warn against overly simplistic adaptationist stories, which are often difficult to test. That said, adaptationist accounts can provide satisfying explanations for some mental disorders. Crucially, adaptationist rationales point to the likelihood that many mental disorders are based on otherwise adaptive functions (25). These rationales can lead to rethinking medicalization or conventional psychiatric nosology by acknowledging the close links between adaptive strategies and pathology (76).

There are also limitations to the adaptationist approach that are external to it. As the logic of evolutionary biology goes, proximate causes acquire explanatory value in so far as they relate to ultimate causes, which are located in evolutionary history. However, in many instances, this history refers to the emergence of human beings in an evolutionary environment of adaptation quite different from our current environments. Evolutionary explanations either appeal to vulnerabilities that arose because of this evolutionary history or focus on discrepancies between past environments, to which we were well-adapted, and current contexts, which pose new challenges (cf. Box 1). New challenges in current contexts, however, are dependent upon socio-cultural features like cultural practices, values and social institutions, whose causal contribution to mental health should be considered (77). Moreover, humans have been co-evolving with our socially constructed environments for millennia (50). Thus, half of the story is missing here. As we will see next, cultural psychiatry provides a concept of mental health consistent with evolutionary thinking, which can provide a mechanistic account of the social systemic embedding of mental health and illness.

### Cultural Psychiatry

Cultural psychiatry acknowledges the influence of multiple processes in establishing the boundaries between the normal and the pathological in biomedical science and clinical practice (70). However, it insists that any perspective must acknowledge context dependence; that is, the influence of socio-normativity of the local cultural contexts. This is crucial to produce definitions of mental disorders that have a grip on clinical practice. Moreover, cultural psychiatry argues that evolutionary history itself is shaped by current cultural concerns and dominant ideologies that may obscure the nature and range of human functioning in health and illness (78, 79). Accordingly, for cultural psychiatry, an evolutionary perspective must consider the social normativity that underlies the use of evolutionary principles to define the normal (functional) and the pathological (dysfunctional) (e.g., the manner in which values of a local ethnomedical practice shapes illness experience and thereby themselves move the boundaries of the normal and the pathological (77, 80).

Cultural psychiatry does not endorse a radical social relativism, which would discount any effort to recognize mental disorders across cultures. Mental disorders are not simply social constructions; they are fundamentally biological. But cultural psychiatry insists that human (neuro)biology is itself fundamentally social—neurodevelopment and adult functioning involve the embedding of the individual in a socially constructed niche and larger interactional systems that are configured by cultural knowledge and practices (81). Recent human evolution has involved cultural-biological coevolution, so that even our thinking about mental disorders in evolutionary terms must engage with the impact of humanly constructed worlds on the structure and function of our brains. Moreover, changes in these social and cultural systems happen faster than evolutionary changes creating potential discrepancies between functional systems and current adaptive demands. The key questions for cultural psychiatry then are not only those that relate to the way in which the social world shapes the experience, definition of, and response to mental disorders, but equally how social contexts and interactions contribute to the underlying mechanisms and developmental trajectories of disorders: that is, how and when mental disorders are constituted by processes that reflect their social systemic embedding.

It is hard to see how one could disagree with the holistic view of mental health proposed by cultural psychiatry. Yet, historically, these claims have been given mostly lip service, as bioreductionism still appears to run deep in psychiatry. To understand the project of cultural psychiatry, we must take a short glance at the recent history of psychiatry and the concept of mental disorder it has employed.

#### Historical Overview of Bioreductionism

The operationalization of diagnostic categories ushered in by DSM-III in 1980 aimed to provide a taxonomy useful for clinical assessment that could also guide research aimed at identifying discrete disorders, each with its own etiology, mechanisms and symptoms (82). Categorical approaches were born from a “biomedical” approach to research and practice that focused on the proximal, biological factors at play and their associated phenotypes (83, 84). The categorical approach of the DSM-III and its successors emerged against the background of already ongoing arguments for a broader *biopsychosocial approach* to assessment (85, 86). On the biopsychosocial view, the illness must be understood in terms of a multilevel hierarchy from molecules to behavior. This affords a conceptual space that accommodates clinical observations in the real-world contexts of disorders (87). However, the hope of characterizing disorders in terms of underlying (biological) mechanisms and the lack of appreciation of the causal effects of social systemic processes has undercut integrative approaches [e.g., (88, 89)].

The current Research Domain Criteria (RDoC) developed by the United States National Institute of Mental Health reflects the emphasis on biological correlates, as it doubles down on neuroscientific research, with the hope of formulating disorders in terms of their (mostly neural) phenotypes and/or measurable (neuro)biological traits (90). Despite the integration of behavioral and phenomenological (e.g., through self-reports) units of analysis, the RDoC framework remains largely bioreductionist (70). In emphasizing biological research, the RDoC relies heavily on evidence derived from animal models. Unfortunately, we have no animal models of many distinctive components of human experiences relevant to mental health and illness, such as narrativity, morality, racism, political violence (91). Reductionism thus is bound to operate with a stripped-down biology that emphasizes brain circuitry over psychological functions and social systemic processes. This makes it difficult for psychiatry to advance its goal of a mechanistic understanding of all the components that make up the gene-brain-person-environment pathway to explain mental disorders. Cultural psychiatry seeks to move toward a concept of mental disorder that remains mechanistic and functional while accommodating culture and context.

#### Toward a Non-reductionist Concept of Mental Disorder

The concept of disorder in psychiatry refers to behavioral patterns that cause psychological distress and functional impairment, and only indirectly to the failure of biological mechanisms. It describes a situation configured at subjective, phenomenological, psychological and social systemic levels (77). Mental disorders are inherently value-laden and shaped by socio-normative causes—e.g., the way we identify the harm resulting from mental ill-health—as much as they are produced by biological causes.

In considering distinctions between health and pathology, cultural psychiatry raises an additional difficulty: namely, giving a scientific account of “harmful”. We need to identify and test the mechanisms by which judgments themselves, understood as objects of language, become consequential for individuals' functioning, wellbeing, social status, etc. Institutional discourse shapes illness experience, which means that we need a functional account of how individual and institutional discourse influence the mind, and how the mind comes to affect institutions. In the notion of 'Harmful dysfunction', the harmful and the dysfunctional must be given equal scientific consideration.

Accordingly, cultural psychiatry defines mental disorders: (i) *pragmatically*, as conditions treated by the discipline of psychiatry, or corresponding local healing practices; (ii) *normatively*, relative to the conceptions of the normal and the pathological given by local medical traditions and practices; and (iii) *ontologically*, as having bodily, psychological, or social systemic causes (30). In employing cross-cultural and ethnographic methods, cultural psychiatry can work out the pragmatic and normative aspects of mental illnesses [e.g., assessing the manner in which individualism in Western culture impacts health and wellbeing (92–95)]. Here we focus on the ontology of mental disorders but recognize the fact that the category of pathology is a moving target influenced by language and culture. Although this remains a challenge, cultural psychiatry captures the moving aspect of ontology using the theory of the looping effects of human kinds, developed by Hacking (19) and (96). We believe that one can leverage the mechanics of looping effects of human kinds to think about a scientific study of the “harmful” in Wakefield's concept of mental disorder.

Kinds are epistemological notions that refer to conceptual classes used to classify, sort or discriminate different objects (19). Natural kinds, for instance, classify objects that undergo *efficient causality*, in the sense that when they are acted upon, those objects conserve the same set of properties. However, objects classified as human kinds, such as mental disorders, do not only undergo efficient causality; they undergo *practical causality—*that is, they change their behavior by virtue of the act of being classified or labeled. This means that kinds are desirable, or undesirable to the people whose behavior fall under their classification (19). It is because they are value laden (that is, they depend on the values assigned to them through social practices) that human kinds are endowed with a causal power different from that of natural kinds. For instance, if N is a natural kind, and Z is an object of the natural kind N, classifying Z as an element of N has no causal effect on Z (19). For instance, if “atom” is a natural kind, calling an “atom” “hydrogen” has no causal effect on hydrogen as an atom. What might change is the way the classifier would engage with hydrogen. The same applies to human kinds (e.g., if I call Denis “autistic”, it will change the way I engage with him). However, while classifying “atom” as “hydrogen” changes only the behavior of the classifier, classifying Denis as autistic also changes Denis' behavior. In contrast to the atom, Denis can become aware of his classification and may change his behavior accordingly. Denis might make less effort, or lose motivation to engage socially because of self-perception and self-evaluation based on his understanding of the classificatory label, or because of his internalization of the stereotypes and social stigma applied by his social partners (97). These proximal interactional effects are, of course, embedded in larger social systemic processes and structures that are major determinants of health and illness (98, 99).

Categories of mental disorders are about people and the criteria they are based on often reference behaviors that are value laden. In turn, our categories of people, their character and values are all culturally shaped (100, 101). This leaves the ontology of any given mental disorder open to change as a function of local cultural changes in norms, conceptual categories, and epistemic practices. For instance, as diagnostic activity and treatments may recognize certain configurations of experience and affliction, clients may access new ways to interpret their experience, thereby yielding corresponding clinical presentations that reinforce the clinician's impression of the validity of the category (30).

Looping effects may entail a shift from one locus to another, such as in cases of somatization, where the affliction may start as a social experience, and then become psychological, bodily, and then social again. Somatization is found across cultures (102) and appears to reflect basic psychophysiological processes that are shaped by culturally specific ways of life and modes of illness experience. These modes of illness experience are culturally patterned ways of expressing bodily and psychological afflictions that reflect cultural models (103). Cultural models are stable discursive and expressive styles of illness experience encoded in individuals' cognitive schema, embodied practices, interpersonal interactions, discourses, and social institutions. It is these cultural models that lie at the interface of individuals and the larger social world to mediate the looping interaction between somatic and emotional/psychological distress (104). Looping effects in cultural models are promising candidates for a mechanistic account of the harmful, in Wakefield's definition of mental disorder as harmful dysfunction.

#### Prospects for an Ecosocial Model of Mental Health

Cultural models point to a concept of mental disorder that recognizes the causal power of social labeling of behavior and experience as *harmful* and aligns more generally with the biopsychosocial approach that recognizes individual cognitive and adaptive processes are embedded in larger social systemic contexts (87). Cultural psychiatry situates the open-ended looping ontology of mental disorders in an ecosocial model of mental health (105), which—much like recent multilevel approaches in psychology (10, 106, 107) and cognitive anthropology (108)—assumes that humans are part of a hierarchically organized, dynamical social ecosystem that includes the brain, the body, and the social and physical environment (109). This means that psychopathological entities may involve dysfunctions not only in their subcomponents (e.g., neuroatypicalities; bodily impairment; and dysfunctional social milieu), but in the system dynamics that bind these components together (110). These dynamics include feedback regulatory processes and mutually causal looping effects that can amplify or self-sustain a psychopathological state (105).

The ecosocial model of mental health gives explicit attention to the systemic embedding of human biology and psychology by drawing links or loops between our self-descriptions (as ill or well) and interactions with the brain, body, and society. It encourages us to consider the multiple forms of social systemic process that give rise to human experience in sickness and in health. In so doing, cultural psychiatry aims to lay bare not only the constructs, norms and constraints that constitute mental disorder as a social reality, but also the cognitive and social interactional processes that may be etiological factors, part of basic mechanisms of psychopathology, and determinants of illness course and outcome. The resultant models of pathology trace the circuits of the mind, which reside not only in the brain but in the social world. However, although well-framed to advance an integrative approach, the ecosocial model of cultural psychiatry, in its current form, remains mainly a narrative description of the mechanisms at the interface between external levels of causation (e.g., socio-material systemic processes) and internal (e.g., brain-based) levels of causation, making it difficult to operationalize in empirically testable models (111). To remedy this, we next consider ways to implement looping dynamics—that undergird the ecosocial model—within the formalism of computational psychiatry.

### Computational Psychiatry

As a domain of clinically applied research in psychiatry, computational psychiatry is primarily motivated by recognition of the shortcomings of current psychiatric nosology in providing diagnostic categories that predict treatment response and outcome and that are linked to mechanistic explanations of disorder (112). However, computational methods also allow us to build models of biological processes that are systemic—that is, they can model networks of many interconnected components and reveal the resulting dynamics. This has proved a powerful approach in systems biology at many levels and, in particular, in efforts to understand how embodied and embedded and extended neural networks can give rise to cognition, behavior, and experience in health and illness.

In this section, we focus on an approach to theory driven modeling in psychiatry known as *active inference* (113–115). Active inference has been proposed as a general framework for understanding the computational processes that underlie cognition and adaptation, which essentially involve prediction of sensory inputs and the effects of actions. This approach understands mental disorders in terms of failure to infer or represent causes of sensations in the world based on Bayesian beliefs, and to act accordingly (112).

Under active inference, mental disorders are defined and modeled in terms of a failure of cognitive functions such as (i) perceptual inference and (ii) adaptive behavior as action planning. Within the terms of our current discussion, active inference can be viewed as seeking an explanation of proximate causes of dysfunctions, where dysfunctions should be understood as suboptimality of perception and action, or Bayesian suboptimality (116). The question active inference asks is: Assuming that the brain operates optimally, how is it that the brain can generate suboptimal behavior? This question is close in nature to that of evolutionary psychiatry: if natural selection optimizes organisms' adaptation, how is it that natural selection can generate suboptimal phenotypic traits (e.g., vulnerabilities)? In both cases, the answer is that suboptimality is the outcome of an optimization process that has “gone wrong” given the developmental, environmental, or social-contextual conditions under which the maladaptation emerged. For evolutionary psychiatry, things can go wrong, for instance, because of a mismatch between the environment of evolutionary adaptation and contemporary social contexts. For computational psychiatry, the optimization process goes wrong when something happens to the cognitive machinery, because of lesions, autoimmune, neoplastic, infectious, or neurodevelopmental anomalies, alterations in neurochemical or neuromodulatory processes, or changes in brain circuitry that may be a result of environmental interactions and learning histories. However, drawing from the arguments of cultural psychiatry, this circuitry may involve systemic processes that extend beyond the brain. We will explore those processes in the Section on Computational Phenotypes Beyond the Brain below.

#### Computational Phenotypes

The theory of active inference allows one to produce computer models of pathological and healthy brain functions to study the effects of various kinds of interventions (mostly psychopharmacological). These models are meant as coarse-grained maps of the brain that translate neuronal architectures (i.e., synaptic connectivity) into parameters, and brain dynamics into belief updating schemes and learning algorithms that update model parameters. Models can be altered in ways that correspond to lesions or interventions and the resultant artificial analogs to behavior can be safely studied *in silico* (117, 118). Of course, the models are inevitably simplified versions of neurobiological systems. When the parameters of these models reproduce psychiatric phenomenology they constitute computational phenotypes: in other words, they provide analogs of pathological neural phenotypes (39). Under active inference, computational phenotypes are statistical generative models that employ Bayesian principles. Crucially, these generative models comprise priors—at many levels—which characterize a particular individual or psychiatric cohort (1, 119). The models are called *generative* because they generate observable consequences from unobservable causes. On this view, the brain is in the game of inverting or fitting a generative model to sensory data; namely, inverting the mapping from causes to consequences to infer unobservable states of affairs in the world from their sensed consequences.

Active inference—in theory-driven modeling psychiatry—assumes that the neural processes underlying perception involve inference *via* the inversion of a generative model [for a discussion of inference under *generative models* and classification under *discriminative models*, see: (120)]. A generative model is simply the joint probability over the causes and consequences that is usually factorized into a likelihood (i.e., the probability of some sensory consequences, given their causes) and prior beliefs (i.e., the prior probability of some causes or hidden states before seeing sensory data).

Active inference assumes that the brain embodies a generative model of its sensory impressions. Sensory impressions correspond to sensory data (e.g., the activity of wavelength selective photoreceptors), and inference corresponds to the inferred cause of the data (e.g., a color). If priors in the generative model are apt to represent the world, the inference about the causes of the data will provide an accurate account of those data in terms of causes, as simply as possible (technically, with minimal complexity; namely, the difference between prior and posterior beliefs). Suboptimal perceptual inference can arise because of a functionally impaired system (e.g., a lesioned brain), or poorly learned priors (e.g., lack of appropriate training experience or a change in circumstances). Generative models instantiated by the brain are highly complex. They are universally composed of hierarchically organized priors (e.g., they contain priors about low-level causal patterns and higher-level abstractions) that are parameterized to reflect the dynamic structure of the world that they are meant to recapitulate.

**Figure 1:**
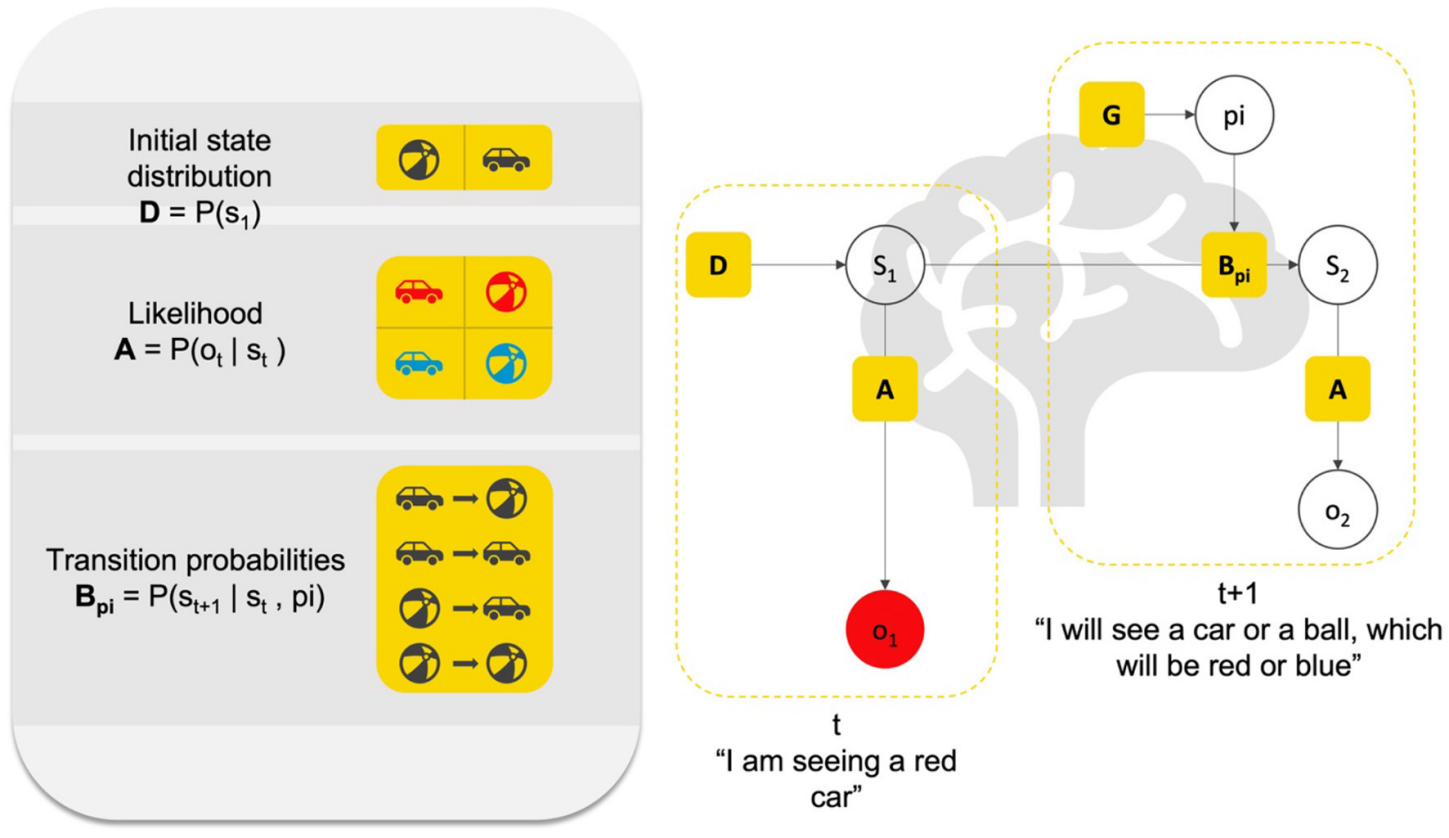
Heuristic description of perception and action as inference. This figure presents a Bayesian graphical model of the relation between (priors and likelihood) parameters that constitute an elementary generative model for action and perception. Here, we can imagine an agent infers the cause “s” of its sensation “o” that it receives at time “1”. This inference is based on a likelihood **A (P(ot|st))** about what generally causes red entries (e.g., is this a car or a ball?), and prior probabilistic knowledge about where the agent currently is in the world (e.g., am I facing a ball or a car?), or initial state **D (P(s1))**. Based on **A** and **D**, the agent can infer s1 (e.g., I am seeing a car). Then, assuming the agent acts based on its perception, it can further leverage knowledge about the dynamics of the world in which it exists, that is, of the transitions between different states of the world (e.g., ball to ball, ball to car, car to ball, etc.), which corresponds to probabilistic knowledge about transitions **B (P(st+1|st,pi)**). Based on **A**, **B**, and **D**, the agent can infer the possible courses of action “pi” to take and the future sensory outcome that it will experience (e.g., red; blue). Representations of causes in the world are hidden states denoted by “s”, representations of allowable policies are denoted by “pi”. Both must be inferred; hence they are in open white circles. Note that “o2” also has to be inferred, since policy selection (i.e., action) involves selecting the course of action toward future sensations. What is known to the agent are the parameters **A, B**, and **D**. Heuristically, perception (small L shape) rests on (implicitly) asking the question: “Given that I see ‘red’ (o1), and knowing that there is a prior probability **(D)** of cars and balls in this environment (e.g., open street), and knowing that both balls and cars can be ‘red’ with a given probability (likelihood **A**), did a car or a ball cause me to see ‘red’?” The answer to that question is the inference in s1. In turn, action rests on asking the question: “Given what I am currently in s1, and knowing the probability of transitioning from s1 to another state (prior about transition **B**), and knowing the sort of course of action I can engage (policy pi), what course of action will lead to the most likely outcome in the future?”. Inferring the current hidden state, the policy and future state and observation is done in a Bayes-optimal fashion, using something called Bayesian belief updating, which can be formulated in a neurobiologically plausible fashion. The inference is always Bayes-optimal; however, if, for instance, the truth about the world is that cars are never blue, and if the agent believes that there is a high probability that cars are blue, this may lead to false perceptual inference. In short, the agent must have the right priors to provide an optimal account of her sensed world.

Inferring the causes of sensations is but one component of the overall task that the brain has to accomplish. The other key task is to select actions that make inference as efficient as possible. Within the context of modeling brain functions, a generative model will include prior beliefs about transitions between states of the world (e.g., moving from “my side of the street” to “the other side”), given allowable actions (e.g., “go forward;” “go backward,” etc.). The imperatives for action selection are the same as those for perceptual inference; namely, to maximize the marginal likelihood of sensory data, under the generative model. The only difference is that for policy selection, this likelihood is averaged over the outcomes predicted under the policy in question. The generative model thus can also infer the best course of action, or action policies (i.e., sequences of plausible actions). In short, active inference assumes that, along with many other functions, perception and action are processes of inference in the brain (for a heuristic description, see Figure 1).

An advantage of using computational phenotypes to study psychopathology is that one is forced to give an explicit mathematical description of the dynamics of the pathological functions to phenotype the disorder (112) (e.g., the neurocognitive process underlying false perceptions, like delusions, and hallucinations). Computational phenotyping of this sort simply entails adjusting the priors of the generative model to maximize the likelihood of a particular subject's behavior or choices (121, 122). Generative models can further simulate psychophysics and neurophysiology (e.g., reaction times and neuromodulatory responses) associated with the hypothesized belief updating mechanisms underlying the pathological function (115). Figure 1 provides a visual description of the way a generative model, or computational phenotype can be used to simulate and study perception and action and decision making under active inference.

#### Computational Phenotypes Beyond the Brain

Reflecting the longstanding interest in modeling neural circuitry, active inference is compatible with the aspirations of the RDoC and shares some of the RDoC's assumptions, namely: that pathology can be understood in terms of circuitry dynamics that adversely affect computational functions, which, typically, subserve adaptive behavior. Both the RDoC scheme and computational psychiatry borrow from a wealth of experimental work that delineates the different ways in which the brain's processing can go wrong. In recent years, in the hope of adopting a more ecosocial perspective on modeling human cognition, research under active inference has attempted to identify the computational-socio-cultural structure of mental disorders. This work has been cashed out in terms of theoretical models and simulation studies of organism-environment interactional behavior (123–129). Enlarging the scope of computational phenotyping, these studies have considered the manner in which organisms leverage their environment to support various cognitive functions and forms of social interaction (e.g., communication, social and situated learning, social conformity, cooperative decision making, joint action, joint attention, etc.) (108).

Conceptually, the ecosocial reading of computational phenotypes is licensed by the fact that the notion of phenotype encompasses levels that reach far beyond the brain (10, 106, 107, 130). For instance, a beaver dam is the product of the beaver's behavior. This behavior determines the beaver's survival and reproductive success; thereby becoming a target for selective processes. The ensuing combination of agents and their niche is known as an “extended” phenotype (131). An agent can also enter into a coalition (or conflict) with its biotic environment, thereby forming a “joint” phenotype, wherein no single party owns the phenotype, such as the health state of a parasite host (132), or, presumably, a shared, patterned cultural practice finessed through cultural evolution driving gene-culture co-evolution (50, 133). Ecosocial computational phenotypes rely on such an extended notion of phenotype to model systems beyond individual brains. This, of course, requires translating the ontology of Bayesian neurocomputation (e.g., prior likelihood and inference) to that of human ensembles.

The general idea behind this translation is simple. Just as the brain engages in inference by inverting a generative model of the cause of its sensations, the environment—and the agents it includes—can be regarded as inferring the cause of the sensory impression the environment receives. From the perspective of the environment, the sensory impressions are the agent's actions. Of course, such an anthropomorphic way of talking about the environment is only meant to set up the computational modeling. For instance, a chair may be viewed as providing a series of action possibilities [also known as *affordances* in ecological psychology (134, 135)], each of which yields different agent and context-dependent probabilities. A seat will have a greater probability to elicit the “sitability” action policy than the “standability” action in, say, a conference room. In this sense, the chair may be viewed as classifying the action “sit” under the category, or the cause for the “agent wanting to sit”. These probabilities are consolidated by histories of agent-environment interactions (e.g., design and construction of the chair, the position of the chair in the room, etc.).

With such a perspective, one can make sense of the many cognitive functions the external world plays for an individual (136) and the manner in which typical and atypical cognition may constitutively depend upon those external functions (124). For instance, we know that perceptual cues guide the acquisition of many cognitive capacities central to normal functioning in social interaction. The production and coordination of perceptual cues such as gestures and uttered narratives guide joint attention during offspring caregiver interaction, and are known to support the acquisition of functions such as folk psychology (which allows a sort of “mind reading” of the states and intentions of others), autobiographical memory, and narrative practices (137–140). The failure of the acquisition of such functions is among the popular—although contentious—explanations of the social symptoms of autism (141, 142). By framing internal and external functions under a single joint phenotype, an ecosocial computational phenotype can explain in a principled fashion (i.e., based on Bayesian principles) the formal relationship between neurocomputational phenomena such as learning and attentional impairments [e.g., (143, 144)], ecological features such as perceptual cues (124), and culturally patterned looping dynamics (145), such as those that characterize interactions between autistic individuals and clinicians or caregivers (97).

The inclusion of non-neural factors in theory driven modeling psychiatry allows one to explain the constitutive role of the environment in mind and cognition. This holds the promise of a mechanistic view of mental disorders that can include the computational role of social context and cultural factors. The same approach can be used to model the embodied nature of cognition: namely, the innermost ecology of mind being the brain in the body, which is embedded in the tool-using, interpersonally communicating individual who participates in a socially constructed (and populated) niche. The Evolutionary, Cultural, and Computational (ECC) model we consider next brings together the ecosocial reading of computational phenotypes presented above with the adaptationist rationale of evolutionary psychiatry.

## Evolutionary Cultural and Computational phenotyping

In the Section Evolution, Culture and Computation in Psychiatry, we reviewed the main motivations and principles of evolutionary, cultural, and computational psychiatry. Our aim was to familiarize the reader with the three approaches and their respective modes of explanation and modeling strategies (see summary Table 1). In this section, we pursue the integration of cultural and computational psychiatry by supplementing the notion of ecosocial computational phenotypes with an evolutionary interpretation of the structure of generative models. This furnishes an Evolutionary, Cultural, Computational (ECC) model of mental disorders. To illustrate how the ECC model might be applied, we will propose a reading of Major Depressive Disorder (MDD) that articulates the manner in which evolutionary and cultural factors can be integrated into a computational narrative to explain symptoms of MDD.

**Table 1:** Modes of explanation and modeling strategies.

**Discipline and mode of explanation**	**Focus with respect to Wakefield's definition**	**Conception of mental disorders**	**Modeling strategy**
**Evolutionary psychiatry** (selectionist account of function and Darwinian rationales)	The concept of dysfunction	Developmentally aggravated vulnerabilities understood as proximate causes shaped by ultimate causes	Darwinian rationales (cf. Box 1)
**Cultural psychiatry** (looping effects of human kinds, impact of self-construal, and ecosocial systemic models)	The concept of the harmful	Behavioral patterns causing psychological distress and functional impairment configured at the subjective level, and shaped by socionormative interactions, and cultural affordances	Ecosocial model
**Computational psychiatry** (active inference and ecosocial phenotyping in theory driven modeling psychiatry)	The concept of dysfunction; potential to model how harm and dysfunction interact	Suboptimal inference of perception and action caused by lesioned or atypically learned prior beliefs	Computational phenotyping

Before we continue, we should highlight an important distinction. Evolutionary and cultural approaches to psychopathology differ from computational psychiatry in that they both start with assumptions about the potential underlying causes of psychiatric phenomena, while the computational approach can remain agnostic^2^. Put another way, theories about psychiatric phenomena in evolutionary and cultural psychiatry aim to account for the specific etiological, phenomenological, and nosological relations between observed symptoms and their underlying causes—and associated syndromes—by drawing from specific accounts of human (pre)history, development, and current social contexts, whereas computational psychiatry can be used to model symptoms by incorporating a variety of possible underlying causes. In that sense, computational psychiatry constitutes a flexible method to analyze psychiatric disorders, rather than a substantive theory of their ontology or etiology.

The upshot of this is that computational psychiatry, or computational phenotyping under active inference, furnishes a way to integrate, within a single coherent and principled framework, a variety of theories about psychiatric phenomena. The ECC approach should not be viewed as a single implementable computational model, but rather, as a description of the variety of priors one could use to parameterize computational phenotypes that conform to the principles of evolutionary and cultural psychiatry. For a simulation study of such a computational phenotype see Constant et al. (16).

### Evolution and Culture in ECC

#### Evolution in ECC

The ecosocial reading of computational phenotypes can be supplemented with an evolutionary interpretation. Internal priors of a generative model can be viewed as targets for selection; they can be studied as (epi)genetic, structural, or *adaptive priors* (146, 147). Adaptive priors are endowed by evolution and have been geared toward adapting the individual to the ancestral environment. They can be contrasted with (empirical) priors which are learned over developmental time *via* experience-dependent neuronal plasticity.

From a modeling perspective, the consequence of this is that adaptive priors will exert a strong top-down influence over empirical priors that can be learned, and thus over behavior and neurophysiology. For instance, our prior preferences for energy rich food can be viewed as an innate prior that will be paired with the learnt empirical prior beliefs about the probability of finding energetic resources in the current environment (148). Such an adaptationist rationale as applied to priors is useful for designing pathological generative models under the views of mismatch theory, constraints and trade-offs argued by evolutionary psychiatry.

#### Culture in ECC

With respect to culture, we have seen that those states external to the generative model—representing the environment, can be modeled in terms of priors and likelihoods, and thus the environment could itself be read as learning about its denizens. This view underwrites the ecosocial interpretation of computational phenotypes. Culture is defined as shared knowledge, practices, values, and institutions that constitute the way of life of a group of individuals or community (30). From a computational perspective, culture may thus be modeled as the calibration (viz. practice) between the priors, likelihood, and agents constituting the environment (viz. institutions) and the priors, likelihood and sensations making up the agents themselves (viz. knowledge and values) (108, 125, 129, 149–151).

The calibration of generative models is mediated by the exchange of sensory cues generated by the environment and actions generated by the agent. Over time, this exchange should attune the generative model of the agent to her environment (108, 126). Cultural models such as those construed by cultural psychiatry (i.e., the stable discursive and expressive styles of illness experience encoded in cognitive schema, practices, and social institutions) may thus be viewed as illness-specific calibrations of agents and their world's generative models, which consolidate through ecosocial looping dynamics.

#### Mechanism and Function in ECC

The ECC considers model parameters that reflect biological (and cultural) phenomena caused by proximal factors (e.g., mechanisms) and ultimate factors (e.g., adaptive functions). This distinction between proximate and ultimate factors, as discussed earlier, is one of the ways in which evolutionary psychiatry tries to understand “why evolution left us with traits that make us vulnerable to mental disorders.” The taxonomy of priors described in this section—i.e., adaptive priors, vs. empirical and environmental or cultural ones—could be misconstrued as promoting a false dichotomy between proximate and ultimate causes: adaptive priors are meant to reflect the species' evolutionary history (its phylogeny), while empirical priors are meant to reflect the way an organism learns its environment over development (its ontogeny). This way of thinking is problematic, however, because it suggests an overly simplistic way to think about adaptation and development.

In particular, the notion of adaptive priors used here might be misread as meaning an “innate” prior, which is a controversial notion that certainly cannot cover many of the kinds of priors relevant to psychiatric disorders. In our model, adaptive priors are distinguished from purely learned, empirical or developmental priors. Historically, the folk concept of innateness has often conflated notions that reflect distinct and often irreconcilable biological realities (152). Those notions include (i) developmental fixity (i.e., the idea that an innate trait is “hard to change”), (ii) species nature (i.e., the idea that an innate trait is “universal”), and (iii) intended outcome (i.e., the idea that an innate trait is “there by design”). Appealing, either implicitly or explicitly, to such a folk essentialist way of thinking in science runs the risk of unjustifiably importing conclusions based on findings in one domain of biology into another disjoint domain (e.g., “because this trait is universal, it must be there by design, and because it is there by design, it will not change over development”) (152). It is precisely these risks that the kind of computational phenotyping proposed here contends with, as it integrates model parameters that are meant to reflect “adaptive” vs. “learnable” traits.

The ECC, however, circumvents the problem of folk essentialism because the notion of an adaptive prior simply refers to a temporal scale of organization relative to a scale of interest. An adaptive prior is one that performs an evolutionary function [for a review of the notion of function, see: (153)] and for that reason, it is reliably transmitted to individuals from one generation to the next (e.g., the hierarchical structure and plasticity of the developing brain) (107). By contrast, an empirical prior is limited to (or learned during) the life span of the system of interest (e.g., a given connection pattern among neurons), and may not be passed on to subsequent generations. Of course, this implies that social systems or niches and cultural contexts that may have temporal duration beyond the life of an individual—and that are passed on exogenetically to the next generation—may also contribute scales of organization relevant to explaining psychopathology (50).

Thus, adaptive priors are typically “hard to change” (for example appear to be developmentally fixed) may simply be “slow to change”; hence, developmental fixity does not suppose a “species' nature”, as that trait may change over phylogenetic time. Universality just refers to the fact that the adaptive prior will be spread across a population for a period extending beyond the individual life span of the members of that population. It denotes the phenotypic synchrony among individuals sharing the adaptive prior within a given (intergenerational) timeframe. Finally, the notion of “design” refers to the evolutionary function of the trait and is manifested by the top-down influence that the adaptive prior will exert on empirical priors (e.g., computationally, for one update at the adaptive level, there might be multiple updates at the empirical level).

At this juncture, it is worth noting that the ECC approach outlined here appeals to a multiscale model of the human brain, called “the hierarchically mechanistic mind”, which explains cognition and behavior by integrating active inference with Tinbergen's four questions in biology (i.e., adaptation, phylogeny, ontogeny, and mechanism) (10, 107). According to this perspective, understanding the computational processes that underlie human action and perception requires an integrative approach that captures the evolutionary, developmental, and real-time dynamics that govern them. By incorporating both adaptive and empirical priors in a single modeling approach, the ECC presents an empirically viable avenue to help researchers unpack the complexities of Tinbergen's four questions. We suggest, therefore, that our modeling approach might not only be of interest to researchers in psychiatry, but also to those in the human and biological sciences more broadly.

### Major Depressive Disorder Under the ECC

Common targets of computational phenotyping include schizophrenia (154), autism (155), and Major depressive disorder (MDD) (156, 157). Evolutionary (Darwinian) and cultural mechanistic explanations have already been proposed to account for the symptoms and syndrome of depression (15, 69).

Computational psychiatry models the core symptoms of MDD (e.g., diminished drive, loss of energy, and anhedonia) in terms of computational failings in the evaluation of long-term utility reward functions, a.k.a. the evaluation of *secondary utility* (156). Secondary utility relates to the value of stimuli whose reward causal structure is complex and spatiotemporally extended (e.g., the reward value of accumulating money). On the other hand, primary, biological, or “hedonic” utility—as opposed to secondary, “anticipatory” utility—relates to reward that is a proxy for reproductive success and survival (e.g., avoiding pain; seeking energy rich food) (156), thereby relating to adaptive priors and preferences that (under adaptationist assumptions) have increased reproductive success in the past. This is consistent with evolutionary approaches to mood disorder arguing for the adaptive value of low mood rather than MDD *per se* (15, 68, 69).

#### Pessimistic Priors

One computational pathway to understanding MDD as a dysfunction of long-term reward evaluation is the acquisition of pessimistic priors that entail biased learning of environmental states. The main function of priors—in a generative model—is to disambiguate the sensory information the system receives, in order to perform successful inference and select adaptive action. For instance, as per our problem of indirect perception, one cannot directly infer the mood of another person solely from the sensory information that person's face affords. Rather, one must take into account some high-level assumptions about the person's behavior over time (e.g., “she is usually a smiling person, but now her smile must mean something different because of what I said yesterday”).

In other words, priors always bias the way we treat incoming information, and consequently, the way one selects action toward future sampling of the environment (e.g., “perhaps I should avoid talking to her as I'm sure she will reject me”). In MDD, priors biasing such model-based decision making are priors that tip the balance toward pessimistic inference, thereby leading to systematic pessimistic thoughts (a.k.a., a negative thinking bias (158). For instance, MDD patients form negative sentences more frequently and faster than healthy controls, when presented with optimistic and pessimistic options (e.g., in the scramble sentence test) (159, 160). As we will see next, pessimistic thoughts may interact with depressive rumination, and lead to the downward depressive spiral of negative expectations and self-evaluation, anhedonia, social withdrawal, and the suppression of reward-approach behavior characteristic of MDD. This is explained in terms of the autodidactic installation of pessimistic priors.

#### Reinforcing Pessimistic Priors

Many symptoms of depression are commonly experienced by healthy individuals and become a target for psychiatric MDD diagnosis only when they become enduring and lead to clinically significant functional impairment. Therefore, any account of depression should explain the maintenance of MDD symptoms over time. Another role of priors is to guide attention toward sensory cues deemed informative, given these same priors (161), a.k.a., *self-evidencing* (162). Explicitly engaged, or endogenous attention, for instance, can be viewed as a form of internal action (163–166) that assesses the relevance of information, sometimes in a biased fashion (167, 168). In MDD patients, aversive events invoke more recurrent and persistent cognitive processing. For instance, depressed patients gaze longer at negative stimuli: i.e., stimuli or information about negative outcomes (169) and spend more time examining them (170). They also report less positive emotion in response to positive images and more arousal to aversive images (171). Sustained endogenous attention over negative stimuli suggests that aversive events are considered informative, that is, disambiguating with respect to pessimistic priors (156).

Recurrent sampling of negative information necessarily entails reduced sampling of positive information (156); the sampling of information being one of the two ways in which one learns and update priors—our bias that drives appraisal of the world (the other being the pruning, or synaptic homeostasis, that underlies structure learning [see: (172, 173)]. Ongoing learning based on negative information is characteristic of the inability to inhibit rumination, defined as the tendency to focus on one's depressive state, along with the causes, meanings, and consequences of one's depression (174). Interestingly, rumination is often motivated by the belief that ruminating will bring insights into how to solve the cause of rumination (175).

The maintenance of MDD symptoms may be explained by the looping effect that underlies the autodidactic learning of pessimistic priors, when considered from the point of view of the computational machinery of the brain embedded in the social world. The loop is simple: pessimistic priors bias attention and learning, which biases active sampling toward rumination and exogenous negative information that confirm the pessimistic prior (i.e., self-evidences it), thereby leading to the consolidation of this pessimistic prior over time (i.e., minimization of uncertainty based on information that confirms the prior) (156). Exogenous negative information propagates in the social world through public discourse as depression becomes an increasingly popular diagnostic label, and characteristic idioms of distress are used by sufferers to frame their experience and guide their attention toward that which conforms to these idioms (176). In so doing, institutionally sanctioned negative exogenous information shapes the way one attends to one's own experience, body, and sensations, thereby reinforcing those priors' beliefs about one's illness.

Indeed, depressive patients are able to leverage and apply emotion regulation strategies to tackle their affliction when they are instructed to do so, but have difficulties selecting such strategies on their own (177). This speaks to the role of the social environment in the maintenance of MDD. It further speaks to the need to model computational looping effects of depression under ECC, not only in terms of learning and action selection dynamics in the generative model, but also in terms of environmental dynamics that feed back into learning to influence subsequent action selection.

#### Pessimistic Priors and Adaptive Priors

We have seen that one of the general mechanism that underwrites MDD may be the maintenance and reinforcement of a pessimistic prior. From a behavioral point of view, the sampling of negative information and rumination reinforces the pessimistic prior. In return, the pessimistic prior further orients the person toward actions that will sample negative information, which accounts for the downward spiral characteristic of MDD. From a cultural point of view, the spiral may be consolidated through pathological cognition. This process is driven by endogenous and exogenous attention: because of pessimistic beliefs, the person attends to negative stimuli, and in return, negative stimuli that confirms the pessimistic beliefs become increasingly available in her environment, social niche or cultural context. In effect, the diagnostic category becomes an organizing framework for experience that exerts its own effects in the cycles that constitute depressive cognition (176)^3^. Of course, this is not the only (or main effect) of culture, which also creates social-structural conditions of adversity and modes of adaptation that engender the vicious cycles of depression (178).

From an evolutionary point of view, given the survival value of being able to rapidly attend to potentially threatening information (179), the learning of a pessimistic prior can be further precipitated by a predisposition to seek negative stimuli, or evidence that will confirm the source of such pessimism. This predisposition can be modeled as a prior preference for the source of negative stimuli. This was demonstrated by Constant et al. (16) in a computational study of the pathogenesis of MDD. They simulated a “social” two-armed bandit scenario, in which the player had to decide which of two social partners to visit. Each partner afforded a level of reward from low to high, and an associated level of uncertainty over whether the visit would afford a high or a low reward. This setting was meant to reflect uncertainty in environmental contingencies, corresponding to the changing mood of social partners. At the outset, the synthetic agent performed the task adaptively and learned optimistic beliefs, until an adverse life event—that increased social volatility—perturbed social contingencies. Learned optimistic beliefs then shifted to pessimistic beliefs, as the agent kept receiving low reward when approaching social partners believed to afford high reward. As the simulation unfolded, expected utility went down, and eventually, the agents stopped engaging altogether, thereby evincing severe social withdrawal and low expected utility characteristic of MDD. Crucially, to reach the MDD state, the agent had to be endowed with a fixed prior preference for high social reward that would incentivize her to keep exposing herself to social partners, despite continued negative evidence (or outcomes). From an ECC point of view, the fixed prior preference played the role of an adaptive prior, which, under normal circumstances, fosters social interactions. However, under abnormal circumstances, for instance, when social volatility increases and persists, the same adaptive prior will generate behavior that engenders low mood and eventually MDD. Accordingly, the pathogenesis of MDD in Constant et al. (16) could be read under the mismatch rationale discussed above. Importantly, this computational study exemplifies our ECC approach by showing how evolutionary and empirical priors that reflect current social-cultural contexts can interact to produce generative models, characteristic of psychiatric disorder.

## Concluding Remarks: Toward an Integrative Systemic View of Mental Disorder

In this paper, we have entertained a trialogue between three approaches to psychiatry: evolutionary, cultural, and computational. We have focused on themes central to these approaches, such as adaptationist thinking, looping effects, and generative models in computational phenotyping. We have suggested a way to merge these perspectives under an Evolutionary Cultural Computational (ECC) model that characterizes the extended phenotype of the individual in context. The goal of this exercise was to exemplify an ecosocial computational model of mental disorders that harmonizes the constructs of evolutionary, cultural, and computational psychiatry, integrating their respective views into a systemic model.

While we believe the ECC approach provides a framework for integrating diverse perspectives in psychiatric theory and research, it has a number of important limitations. The ECC approach puts few constraints on theory building and an ECC computational model will only be as accurate as the evolutionary and cultural models that inform it. Computational models are technically challenging and require specific training to conduct analyses, which may not be part of the skill set of those with the requisite expertise in evolutionary or cultural psychiatry. As a method of building hypothetical models, the validity of ECC cannot be directly tested. Ultimately, its validity rests on its scientific and practical utility of generating new models, which make testable predictions. The performance of any one ECC model can be compared against competing models and real-world data to confirm or refute simulation outcomes. Translating computational models to psychiatric practice presents its own challenges, which might be met by developing diagnostic and assessment tools that allow practitioners to use client data to predict the course of illness in different social contexts or under different treatment conditions.

Despite those limitations, we believe that there are several ways in which the proposed ECC model can contribute to psychiatric theory, research, and practice. Active infererefinence models in computational psychiatry are meant to function as heuristic descriptions of the brain. Based on these heuristics, one can simulate pathological behavior and test, *in silico*, various interventions that mimic the effects of pharmacological agents, psychotherapy, social interventions, or other treatments on model parameters to examine the potential efficacy of this treatment to return the agent to “normal” functioning. Such modeling can suggest the sensitivity of illness trajectories to particular types of intervention and the potential interactions among multiple interventions.

Because the ECC model considers evolutionary and cultural parameters, *in silico* testing of an ECC model may provide new insights into the potential efficacy of interventions in more ecologically valid contexts [e.g., for a simulation study applying an ECC model to depression, see: (16)]. ECC phenotyping methods can be used to simulate specific kinds of suboptimal perceptual inference (e.g., the misinterpretation of a social partner's intention) that may be associated with psychiatric disorders by considering the influence of parameters reflecting the neural, developmental, evolutionary, and social dimensions of a phenotype. ECC phenotyping methods can also be used to identify clinically relevant phenotypes by fitting simulations to large datasets harvested from a range of different contexts, including: data drawn from interactions in shared environments such as social media platforms (which would reflect the manner in which people engage in a shared generative process); data drawn from psychophysics (e.g., eye tracking and response time data); and imaging or EEG data (which would reflect the impact of individuals' generative models on behavior). Using standard methods for Bayesian model comparison [e.g., Bayesian model reduction (180, 181)], researchers could compare ECC phenotypes in terms of their model evidence, each emphasizing different components of the phenotype.

Finally, returning to the problem of disciplinary boundaries discussed at the outset, the ECC model—understood as a multidisciplinary platform to integrate diverse approaches to psychiatric phenomena in the same computational model—could allow practitioners with various backgrounds to see how their perspectives can connect and converge; thereby enriching each other's ways of thinking about psychiatric disorders. Indeed, the goal of the ECC model is to allow researchers and clinicians to consider how phenomena like adaptation can contribute conceptually to an understanding of culture, and *vice versa*, that is, how cultural context and meaning shape the exigencies and outcomes of adaptation in health and illness. Clearly, the human mind involves highly complex processes that incorporate nested levels of organization and boundaries that reflect our cultural co-evolution and varied forms of social life. If we are to come to grips with the difficulties in adaptation and functioning that are the domain of psychiatry, we must develop tools that capture such complexities.

## Data Availability Statement

The original contributions presented in the study are included in the article/supplementary material, further inquiries can be directed to the corresponding author.

## Author Contributions

AC wrote the first draft. PB, LK, and KF assisted each in turn in the revision and modification of the first draft. All authors contributed to the article and approved the submitted version.

## Funding

This work was supported by the Australian Laureate Fellowship Project, A Philosophy of Medicine for the Twenty-First Century (Ref: FL170100160) (AC), by a Social Sciences and Humanities Research Council Doctoral Fellowship (Ref: 752-2019-0065) (AC) and by the Wellcome Trust (Ref: 088130/Z/09/Z) (KF) and the Canada First Research Excellence Fund (CFREF), Healthy Brains for Healthy Lives, Canadian Framework for Brain Health (LK).

## Conflict of Interest

The authors declare that the research was conducted in the absence of any commercial or financial relationships that could be construed as a potential conflict of interest.

## Publisher's Note

All claims expressed in this article are solely those of the authors and do not necessarily represent those of their affiliated organizations, or those of the publisher, the editors and the reviewers. Any product that may be evaluated in this article, or claim that may be made by its manufacturer, is not guaranteed or endorsed by the publisher.

## Footnotes

^1^In this paper, we use a folk concept of mechanism—of the sort that any typically trained psychiatrist would have in mind in clinical case formulation. In this context, a mechanistic approach may be loosely defined as one that analyses the causal processes that produce a given (psychiatric) outcome, through reference to constituent components and their interactions. There is an important debate in the philosophical literature about the precise nature of mechanism (3–5). Our previous work on the embodied and situated human brain is aligned with a neo-mechanistic perspective in the philosophy of science [e.g., (6–9)], which explains the properties, functions, and behavior of a system by examining the properties and activities of its various subsystems and their interactions. Here, a mechanism can be described as a structure (or a stabilized process) within a system that performs a function via its component parts, their various operations, and their organization, thereby contributing to global function in one or more ways. According to previous work on the hierarchically mechanistic mind (10) consistent with the present approach, the human phenotype is produced by causal mechanisms that span multiple spatial scales (e.g., genes, cells, tissues, organs, the body, and the broader social and physical environment), as well as temporal scales (ranging from evolutionary/intergenerational processes, through to developmental influences, and mechanisms that operate in real-time biopsychosocial contexts) (11). In short, this multilevel theory describes human phenotypes in terms of the biopsychosocial processes that operate within and across different spatiotemporal scales, and in this sense, it is both mechanistic and hierarchical.

^2^To the extent that computational modeling is based on a theory of how the brain works (or, at another systemic level how social interactions work), it may also make assumptions about causality. However, computational modeling can be used simply to provide a model of observed relationships (i.e., input/output mappings) with no presumption that the model describes the actual mechanisms mediating those relationships). When modeling attempts to describe how the brain actually works, the computational model will usually be underwritten by specific evolutionary or socio-cultural accounts of function which have direct consequences for and constraints on the model. The distinction overall is between computational models of the brain as specific ontological theories and computational models as a generic toolkit to capture dynamics that may be instantiated in diverse ways on different substrates. This is precisely one of the ongoing debates around active inference in modeling psychiatry. How much does such modeling assume or entail about the brain as opposed to simply being a flexible framework for (re)describing observed relationships? This is an open debate that we cannot settle here but we note that there are a range of possibilities related to the theoretical or empirical basis for structural relationships that are built into the computational model (rather than those than emerge by virtue of its dynamics).

^3^This kind of looping effect provides a key illustration of how a social-cultural perspective enriches the evolutionary computational model.
